# Effect of erosive and abrasive stress on sealing ability of different desensitizers: *In-vitro* study

**DOI:** 10.1371/journal.pone.0220823

**Published:** 2019-08-01

**Authors:** An-Na Choi, Il-Seok Jang, Sung-Ae Son, Kyoung-Hwa Jung, Jeong-Kil Park

**Affiliations:** 1 Department of Conservative Dentistry, School of Dentistry, Pusan National University, Dental Research Institute, Yangsan, Korea; 2 Department of Microbiology, School of Natural Sciences, Pusan National University, Busan, Korea; National Taiwan University, School of Dentistry, TAIWAN

## Abstract

This *in vitro* study examined the sealing ability of different desensitizing agents under a chemo-mechanical stress condition. For the study, a total of 144 extracted, caries-free human third molars were used to produce 1 mm-thick dentin discs. The specimens were divided randomly into four groups: Superseal (SS), Gluma (GL), Gluma Self-etch (GS), and Tooth Coat (TC). For each group, the permeability was measured before and after applying the desensitizer, after being exposed to Coca Cola for 5 minutes, and after 3150 strokes of a brushing abrasion. The decrease in permeability after the erosive and abrasive stress was analyzed by ANOVA and Tukey post hoc test. As a result, the dentin permeability decreased significantly for all desensitizers immediately after application (p < 0.05). SS and GS showed a significant difference in permeability reduction observed immediately after application and after acid action with Coca Cola (p < 0.05). After brushing abrasion, the permeability reduction decreased significantly for all desensitizers tested in this study (p < 0.05). TC showed the largest decrease in dentinal permeability compared to that of the other desensitizers and the differences were significant after brushing abrasion (p < 0.05). All tested desensitizers were effective in reducing dentin permeability. The behavioral characteristics under erosive and abrasive stress varied according to the products used. TC exhibited excellent sealing ability among the other desensitizers.

## Introduction

Dentin hypersensitivity (DHS) is a widespread condition that can cause inconvenience to patients’ lives with a prevalence ranging from 3% to 98% [1]. This range of epidemiologic data may be attributed to differences in the study design including the types of assessment protocols, inclusion criteria, or regional variations. DHS is characterized by short, sharp pain arising from the exposed dentin in response to external causative stimuli including thermal, tactile, evaporative, osmotic, or chemical, which cannot be ascribed to any other form of dental defect or pathology [2]. All causes of dentin exposure, including loss of enamel due to occlusal wear, over-zealous tooth brush abrasion, erosion, abfraction, parafunctional habits, and loss of cementum due to gingival recession, periodontal disease, root planning, and periodontal surgery, could lead to DHS [3,4].

The most widely accepted mechanism of DHS is the hydrodynamic theory proposed by Brännström and Astron in 1964 [5]. This mechanism is based on the capillary flow dynamics of fluid-filled dentinal tubules [6]. When physical stimuli are applied to exposed dentin, the tubular fluid volume will expand or contract and form inward or outward fluid shifts through capillary action [7]. The hydrodynamic forces resulting from the rapid displacement of fluid excite the mechanoreceptors in the A-δ pulpal nerve fibers surrounding the odontoblasts in the superficial pulp [8].

A number of desensitizing agents are available for either in-office or over-the-counter (OTC) applications [9]. These are classified into two main types according to their mechanisms of action: “nerve blocking” and “tubule occlusion” [10]. Potassium-based (chloride, citrate, and nitrate) products reduce the pulpal sensory nerve activity by direct ionic diffusion through the increased potassium ion concentration [11,12]. Tubule blocking agents such as potassium oxalate, fluoride, calcium phosphates, arginine-calcium carbonate, or biomimetic mineralization materials, lead to a decrease in the functional diameter of the tubules through the formation of insoluble precipitates within them. Hydroxyethyl methacrylate (HEMA), with or without glutaraldehyde, occludes the tubules with precipitated plasma proteins in the dentinal fluid, thereby reducing the dentin permeability [13–15]. Varnishes, resin-modified glass ionomers, or dentin adhesives also reduce the dentin permeability by sealing the exposed dentinal tubules [16,17].

Although a number of desensitizing agents have been reported to be effective in reducing the dentin permeability [18–20], their efficacy is likely to be short-lived [21]. Owing probably to the fact that many desensitizing agents do not adhere to the dentin surfaces [18], they inevitably suffer from thermal, erosive, and abrasive stress in the oral cavity. Newly developed desensitizing agents are being introduced to the market unceasingly; however, their behavioral characteristics under the challenge of erosive and abrasive conditions have not been sufficiently investigated. Therefore, this *in vitro* study evaluated the sealing ability of different desensitizing agents under chemo-mechanical stress conditions using a permeability measurement system. The null hypothesis tested was that neither the type of desensitizing agents nor chemo-mechanical stress would affect the permeability of the dentin.

## Materials and methods

### Materials

Four desensitizers were used in this study: Superseal (Phonix dental, Fenton, MI, USA), Gluma desensitizer (HeraeusKulzer GmbH, Hanau, Germany), Gluma Self-etch (HeraeusKulzer GmbH, Hanau, Germany), and Tooth Coat (Osstempharma, Pusan, Korea). Table 1 lists the compositions and application methods of the desensitizers.

**Table 1:** Compositions and application methods of the desensitizers.

Material	Abbreviation	Composition	Application method
Superseal	SS	Oxalic acid Potassium salt	The tooth surface was rinsed with water and air-dried. Agent was applied, left undisturbed for 30 s, and gently air-dried.
Gluma	GL	HEMA^a^ Glutaraldehyde Purified water	The tooth surface was rinsed with water and air-dried. Agent was applied, left undisturbed for 60 s, gently air-dried until the fluid film disappeared, no longer shiny, and further rinsed with water again.
Gluma Self-etch	GS	HEMA Glutaraldehyde Acetone, water Photoinitiator 4-MET^b^	The tooth surface was rinsed with water and air-dried. Adhesive was applied three times, left undisturbed for 20 s, gently air-dried until no movement was observed, and further light-cured for 20 s.
Tooth Coat	TC	5% NaF^c^ Hydrogenated rosin PVAc^d^	Tooth surfaces were rinsed with water and air-dried. Agent was applied using a disposable brush. Because the agent sets when in contact with water or saliva, it should remain undisturbed on the teeth for 5 min.

^a^HEMA: 2-hydroxyethyl methacrylate

^b^4-MET: 4-methacryloyloxyethyl trimellitic acid

^c^NaF: Sodium fluoride

^d^PVAc: polyvinyl acetate.

### Tooth preparation

A total of 144 extracted, caries-free human third molars within one month of extraction were used. This study was approved by the Institutional Review Board of Pusan National University Dental Hospital (IRB, PNUDH-2017-030). The teeth were disinfected with 0.5% chloramine T and stored in a physiological saline solution at 4°C until used. The crowns of the molars were sectioned with a water-cooled diamond disc (Accutom-50, Struers, RØdovre, Denmark) perpendicular to the long axis of the tooth at 2 mm below the deepest occlusal pit or central groove to remove all the occlusal enamel and superficial dentin. The second section was made in the same plane at 1 mm below the first section to produce 1 mm thick dentin discs. This thickness is sufficiently permeable to allow the screening of desensitizing agents through the *in vitro* model [22,23]. The specimens were immersed in 0.5 M EDTA (pH 7.4) (Merck, Darmstadt, Germany) for 1 min and ultrasonicated for 2 min to remove the smear layer on both sides of the discs before a final rinse with deionized water. Specimens with baseline dentin permeability values between 2–5 μL min^-1^ were selected. Using this criterion, 120 out of the original 144 dentin discs were used for the experiment.

### Experimental design

**Fig 1:**
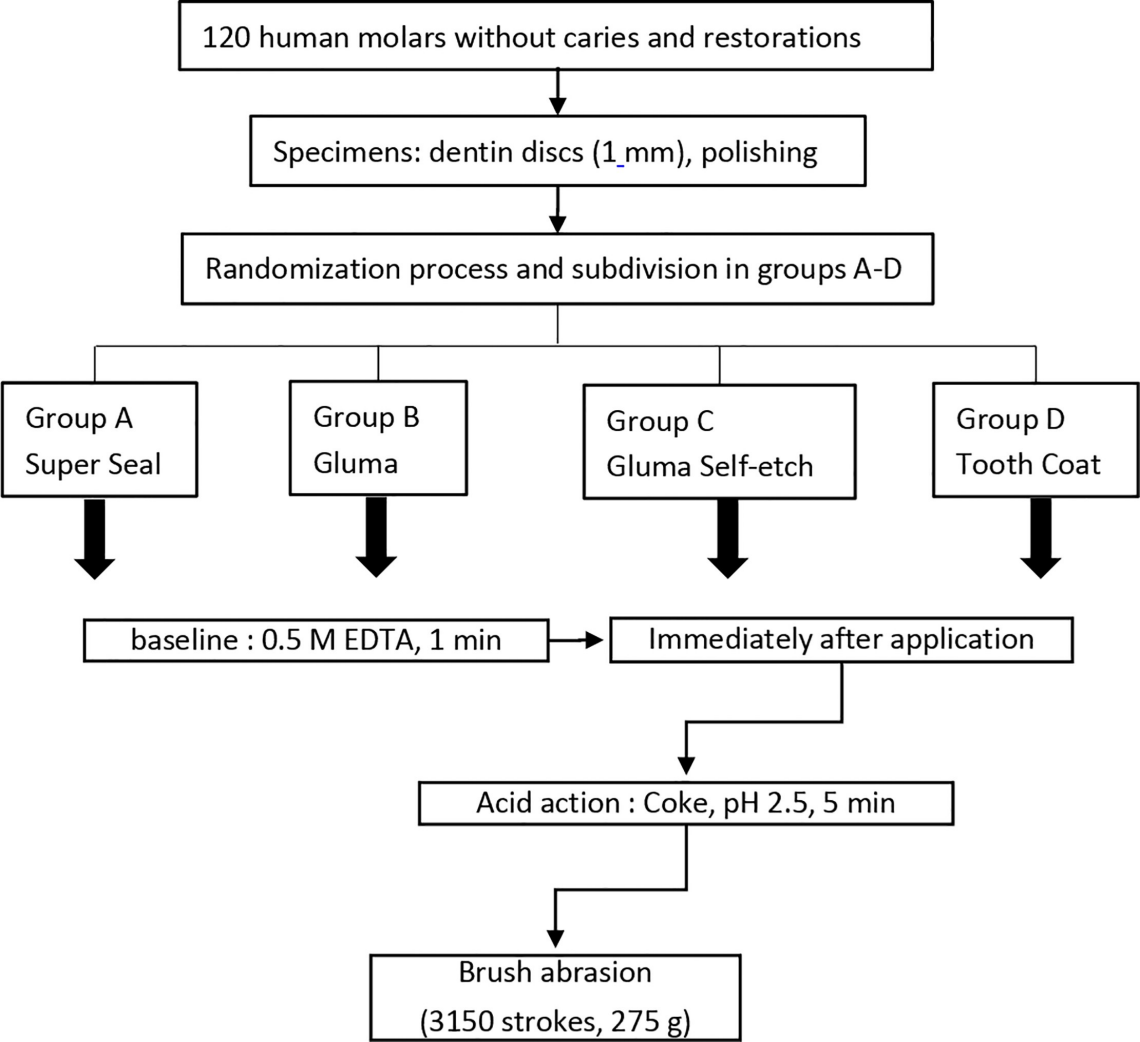
Summary of the experimental design to prepare dentin specimens for dentin permeability measurements and SEM analysis.

Fig 1 presents the experimental design. The 120 specimens were divided randomly into four groups (each group: n = 30): Superseal (SS), Gluma (GL), Gluma Self-etch (GS), and Tooth Coat (TC). All the desensitizers tested in this study were applied according to the manufacturer’s instructions. The specimens which were treated with 0.5 M EDTA (Merck, Darmstadt, Germany) for 1 min served as controls. The specimens were then exposed to Coca Cola (Coca Cola, Coca Cola GmbH, Berlin, Germany) for 5 min and the permeability was measured. Subsequently, 3150 strokes of brushing abrasion were applied with a slurry of synthetic saliva and fluoride-free toothpaste (Sensodyne C, GlaxoSmithKline Consumer Healthcare GmbH &Co. KG, Hamburg, Germany) to simulate 3 months of tooth-brushing. An automatic brushing machine (Brushing Machine Tester, HanGil Technics, Hwaseong-Si, Korea) was used for brushing abrasion with a loading mass of 275 g. Finally, the permeability was measured again. The permeability measurement was performed as detailed below.

### Permeability measurement

**Fig 2:**
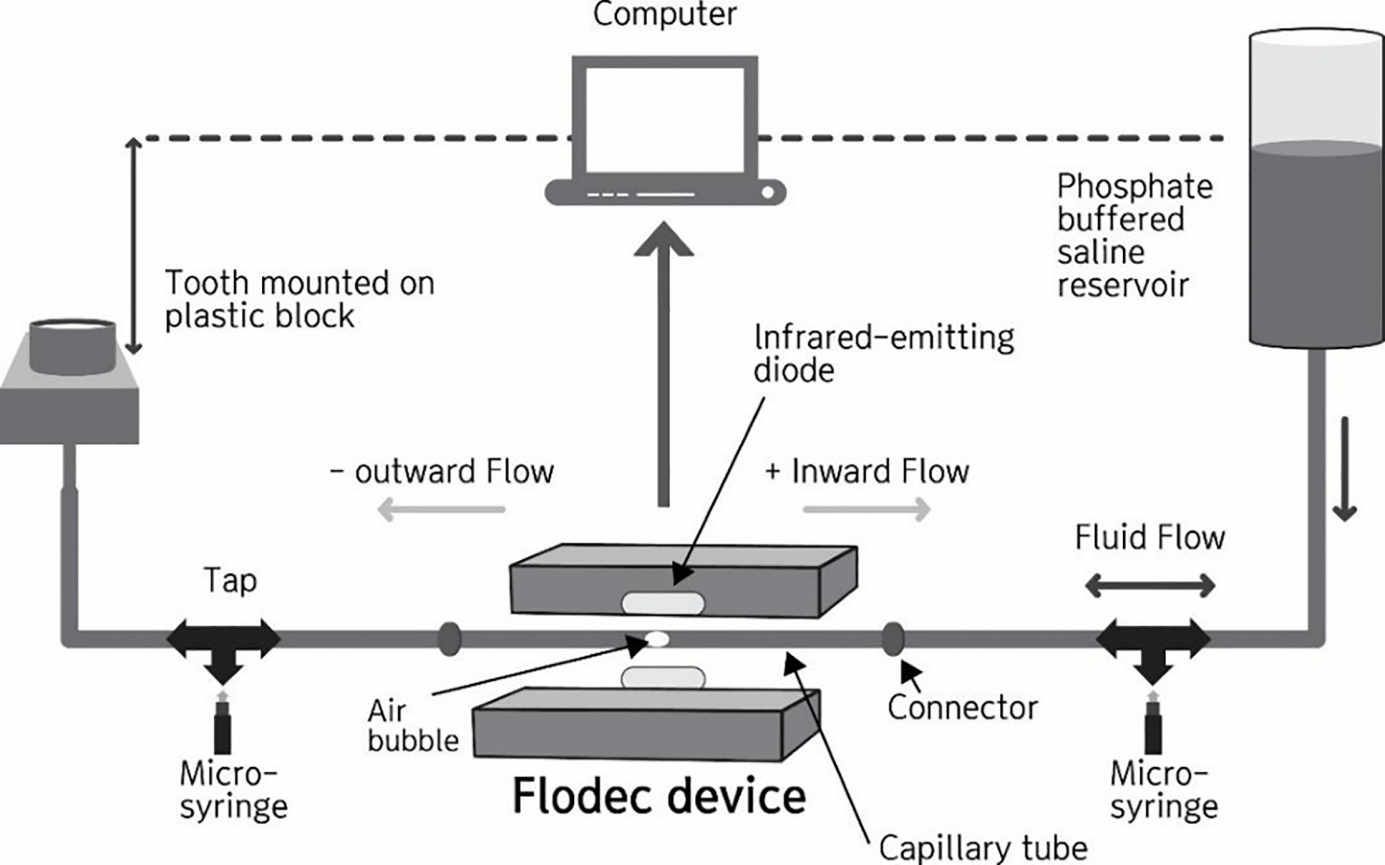
Schematic diagram of permeability measurement system.

The rate of fluid flow through a dentin specimen was measured using a THD03d device (Odeme, Luzerna, Brazil), as illustrated in Fig 2, which follows the movement of a tiny air bubble as it passes down a 0.6 mm diameter glass capillary located between a water reservoir under 140 cm (2 psi) of water pressure and the dentin specimens. A physiological pressure (2 psi) was selected to simulate the human pulpal pressure, as reported by Zhang Y et al. [22]. An infrared light source passes through the capillary and is detected by a diode, allowing the unit to follow the progress of the air bubble along the length of the capillary. The linear displacement is converted automatically to a volume displacement per unit time, from which the instantaneous volumetric flow rate is calculated and logged into a spreadsheet. The flow was measured until a steady-state was reached, typically 0–3 min; the flow was then measured for at least 2 min. One datum was taken every second, resulting in at least 100 readings under each condition. The permeability is expressed as the fluid flowrate in μLmin^−1^.

### Scanning electron microscopy (SEM) analysis

Two specimens per stage of the groups were selected randomly for SEM analysis. The specimens were washed, air-dried, and mounted on an aluminum stub. After coating with a thin layer of gold/palladium (Sputter Coater 108auto, Cressinton, Watford, UK) in a sputter coater, the surfaces of these specimens were scanned and examined by SEM (JSM-6480LV, JEOL, Tendo-shi, Japan). The presence of any dentinal surface alteration, precipitation, or debris was detected. Representative SEM images were judged by the examiner based on the frequently observed appearance of the specimens to represent each experimental group.

### Statistical analysis

The post-treatment of the values is expressed as a percentage of the baseline values, allowing each specimen to serve as its own control. Statistical analyses were used to examine the decrease in permeability as a percentage (%). Comparisons among the four desensitizing agents over chemo-mechanical stress were analyzed using a two-way ANOVA and Tukey’s post hoc test (p < 0.05). A one-way ANOVA and a Scheffe’s multiple comparison test (p < 0.05) were also performed to compare each condition individually, regardless of the agents or chemo-mechanical stress conditions. The SEM images were evaluated only qualitatively.

## Results

### Permeability measurement

Table 2 presents the results obtained by two-way ANOVA for the percentage decrease in permeability. The study was adequately powered for both factors: the desensitizer and chemo-mechanical stress condition (over 95%; p = 0.05). Two-way ANOVA indicated that the factors, “desensitizer” (p < 0.0001) and “chemo-mechanical stress” (p < 0.0001), along with their interactions (p = 0.019), had a significant influence on the permeability.

**Table 2:** Results of two-way analysis of variance.

Source	*df*^a^	Type III sum of squares	Mean square	F	P
Desensitizing systems (DS)	3	6250.88	2083.63	10.22	< .0001
Aging process (AP)	2	27984.60	13992.30	68.61	< .0001
DS * AP	6	3178.42	529.74	2.60	0.02

^a^*df*: degree of freedom

**Fig 3:**
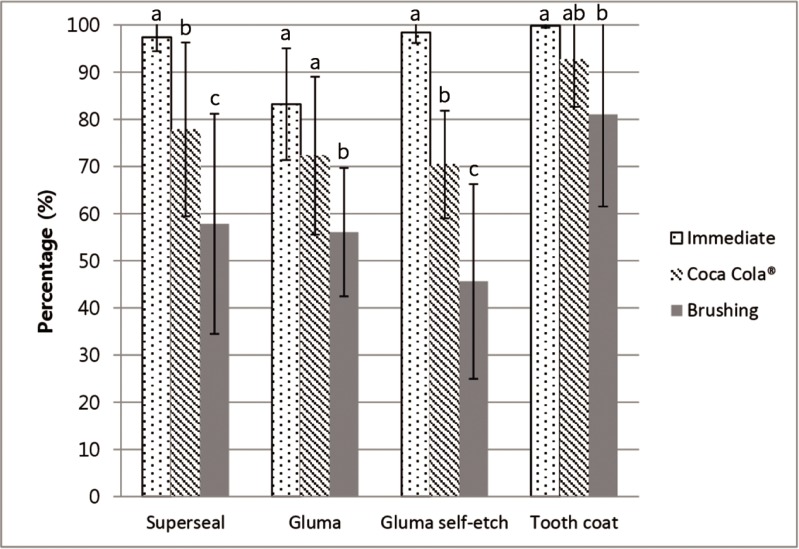
Mean (SD) percent reduction (%) in permeability of the four desensitizing agents.

Table 3 lists the multiple comparisons obtained by one-way ANOVA for the percentage decrease in permeability. The permeability before applying the desensitizer indicates the baseline values, and was used as a control to compare the changes in permeability throughout the experimental process. The dentin permeability decreased significantly for all desensitizers immediately after application (p < 0.05). SS and GS showed a significant difference in permeability reduction immediately after application and after acid action with Coca Cola (p < 0.05). After brushing abrasion, the permeability reduction was reduced significantly for all desensitizers tested in this study (p < 0.05). TC achieved a greater reduction in dentinal permeability than the other desensitizers, and the statistical differences were significant after brushing abrasion (p < 0.05). Fig 3 presents graphically the changes in the percentage decrease in permeability in the experimental process by the different desensitizers.

**Table 3:** Mean (SD) percent reduction (%) in permeability of the desensitizers.

	Dentin permeability reduction (%)		
Group	Stage I^1^	Stage II^2^	Stage III^3^
Superseal	97.41 (2.95)^Aa^	77.83 (18.43)^ABb^	57.84 (23.33)^Ac^
Gluma	83.21 (11.83)^Ba^	72.27 (16.74)^Aa^	56.07 (13.59)^Ab^
Gluma Self-etch	98.42 (2.27)^Aa^	70.42 (11.40)^Ab^	45.61 (20.64)^Ac^
Tooth Coat	99.86 (0.44)^Aa^	92.65 (9.99)^Bab^	81.00 (19.49)^Bb^

^1^Stage I: immediately after application

^2^Stage II: after Coca Cola immersion

^3^Stage III: after brush abrasion.

The same letters indicate mean values with no statistical differences. (Uppercase letters = column, lowercase letters = rows).

### SEM analysis

**Fig 4:**
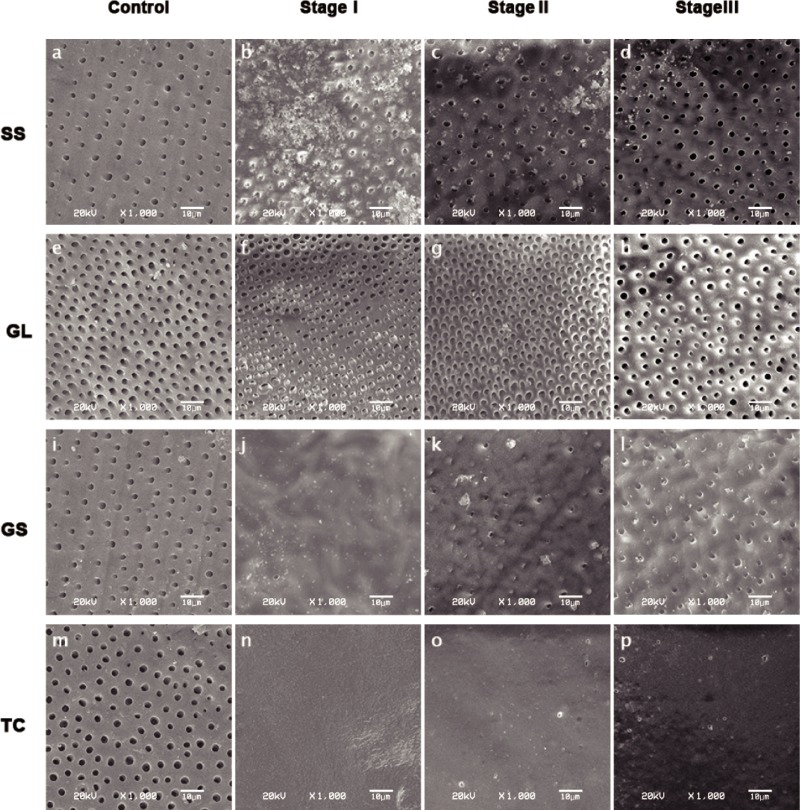
SEM micrograph of experimental groups. Stage I: immediately after application; Stage II: after Coca Cola immersion; Stage III: after brush abrasion. SS: Super Seal; GL: Gluma; GS: Gluma Self-etch; TC: Tooth Coat.

Specimens of the control revealed opened dentinal tubule orifices due to the removal of a smear layer (Fig 4A, 4E, 4I and 4M). Different changes in the morphologies of the dentin surfaces were observed according to the groups. Fig 4B and 4C show calcium oxalate crystals inside the tubules and on the dentin surfaces. The granular calcium-oxalate deposits did not form on the surface uniformly (Fig 4B and 4C). Fig 4D shows that there were low deposits on the surface, and the orifices were rarely closed in the tubules. The surface morphology of the specimens treated with GL was not uniform and some dentinal tubules were occluded, some partially occluded, and others appeared open (Fig 4F–4H). Fig 4J shows the adhesive layer covering the surface and some porosity in the adhesive layer of the GS-treated specimens. Some areas with no apparent hybrid layer remaining were also observed (Fig 4K and 4L). The surfaces of the TC group were covered with a homogeneous layer of material without any visible dentinal structures (Fig 4M–4P). Despite the low porosity on some areas of the TC group, no apparent modification was observed after challenging the surface with Coca Cola or brushing abrasion (Fig 4M–4P).

## Discussion

The present *in vitro* study has provided evidence to support that all tested desensitizers are effective in reducing dentin permeability. However, the results showed that the sealing ability of the desensitizers differed significantly according to the erosive and abrasive stress condition. Therefore, the null hypothesis that neither the desensitizing agents nor the chemo-mechanical stress would influence the permeability of the dentin was rejected.

In the present study, direct measurements of fluid flow through the dentin were performed to evaluate the sealing ability of the four desensitizers. Poiseuille’s law states that the resistance to fluid flow through the tubules is inversely proportional to the fourth power of the radius of the tubules [24]. It is assumed that a decrease in the radius of the dentinal tubules will lead to resistance to fluid movement, thereby reducing DHS [24]. Although the precise correlation between the incidence of DHS and dentin permeability has not been established [25], post-treatment reduction of the dentin permeability compared to pre-treatment is a reasonable method to measure the sealing ability of a desensitizer [26–28].

Acidic solutions of potassium oxalate have been used for DHS in clinical dentistry. Potassium oxalate desensitizers, such as Super Seal, react with the ionized calcium and form insoluble granular calcium oxalate, which precipitates both within the dentinal tubules and on the surfaces of the dentin, enamel, and cementum [29–31]. Calcium oxalate is an ionic compound with the chemical formula CaC_2_O_4_ and a salt of oxalic acid, which is highly insoluble at neutral pH (7.0). In the present study, the precipitation of calcium oxalate crystals was observed immediately after application in the SEM image (Fig 4B). The permeability reductions that have been reported previously for oxalates were up to 98% [32,33]. The mean value of 97.41% measured immediately after application obtained in the present study is consistent with the published results, but the permeability was affected by the Coca Cola and brushing abrasion. The mean value of 77.83% after the acid action of Coca Cola was significantly lower than that measured immediately after application. The results of the present study suggest that the low pH of Coca Cola might dissolve the calcium oxalate crystals formed in the dentinal tubules and on the surfaces. This result was also consistent with an additional study, which found that the solubility of calcium oxalate crystals located inside the dentinal tubules is sensitive to pH [34]. In addition, the mean value of permeability reduction measured after brushing abrasion was significantly lower than that measured after acid action. These results indicate the susceptibility of the calcium oxalate deposits to abrasion, and also to erosion. It is thought that calcium oxalate crystals might not form a tight chemical association with the tooth substrate, but simply precipitate into the dentinal tubules or surfaces. Nevertheless, the permeability reduction remaining after acid action or brushing abrasion reflects the possibility that calcium oxalate crystals located deeper inside the dentinal tubules could disturb the fluid transudation across the tubules. From the present study, SEM could only observe the precipitation that occurred solely along the outer surface of the dentin.

Gluma is a glutaraldehyde-based HEMA formulation that contains an aqueous solution of 5% glutaraldehyde and 35% HEMA. Glutaraldehyde is an amine-reactive homo-bifunctional cross-linker that reacts with serum albumin in the dentinal fluid and precipitates plasma proteins by coagulation inside the tubules [35]. This precipitation mediates the second step of the polymerization of HEMA, leading to physical blockage of the tubules [36]. Although there are many sources of proteins such as collagenous and non-collagenous proteins from dentin, not using the phosphate-buffered saline (PBS) or simulated dentinal fluid could negatively affect the mechanism of precipitation of proteins by glutaraldehyde in this *in vitro* study. Nevertheless, we stored specimens in deionized water to exclude inadvertent sedimentation in the glass capillary, which would impair the consistency of the results. The permeability reduction results reported previously for glutaraldehyde-based solutions range from 28.0% to 77.0% [37–39]. The mean value of 83.21% obtained in the present study with Gluma measured immediately after application was higher than those obtained by other studies. The variation of permeability reductions might be due to the different experimental designs and execution. The survey of Brunton et al. [40] revealed that GLUMA possesses low acid dissolution resistance, which may be explained by its hydrophilic components that can be easily removed or degraded in an acidic environment, such as in the case when the specimens were immersed in Coca Cola for 14 days. The permeability reduction after acid action, however, was not statistically different from that measured immediately after application in the present study. This discrepancy might be due to the different duration of acid challenge with an erosive challenge for 5 min in the present study. In addition, previous studies reported the presence of transverse septa in the dentinal tubules formed by the precipitation of plasma proteins derived from the dentinal fluid [41]. Schüpbach et al. observed the intra-tubular septa to a depth of 200 μm [42]. Considering these findings, the multiple layers of protein septa located deep within the tubules might have a potential to maintain the sealing ability of Gluma, even after acid action or brush abrasion under the experimental conditions of this study. Arraies et al. examined SEM images of Gluma and identified a thin layer that covered the dentin specimens [43]. In contrast to this finding, however, a covering layer was not observed in the present study. Guentsch et al. also could not observe a clear layer in the Gluma-treated specimens [15]. These conflicting results might be related to operator-related variables, such as the intensity of brushing motion, air pressure to dry the agents, or a determination of the end-point of air-drying before rinsing with water.

Several studies have confirmed that the topical application of dentin adhesives is effective in reducing DHS [44]. Dentin adhesives can occlude any patent dentinal tubules, leading to a decrease in dentin permeability [45]. Schmalz et al. examined the dentin protection of different desensitizing agents during acid action/abrasion stress and thermocyclic loading *in vitro* and suggested that light-curing agents ensure higher dentin protection [46]. In contrast, Gluma Self-etch had only an immediate effect on reducing the permeability and the effect was reduced significantly in the present study by the acidic action of Coca Cola and brush abrasion. Gluma Self-etch is classified into the 7^th^ generation of dentin adhesives, which combines the acid, primer, and bond in a single bottle based on an all-in-one concept. In general, single step self-etch adhesives contain high concentrations of acidic functional monomers, hydrophilic monomers, such as 2-hydroxyethyl methacrylate (HEMA), water, and/or organic solvents into a single solution [47]. Unfortunately, the residual water in the water-, acetone-, or alcohol-based primers, which evaporates incompletely due to the high surface tension of water [48], reduces the degree of conversion of adhesives [49] and results in a water-filled channel or water trees within the hybrid layer and adhesive layer [50]. Tay et al. reported that single-bottle self-etch adhesives served as a permeable membrane [51]. Hashimoto et al. detected fluid movement across the resin–dentin interface after the polymerization of adhesives [52]. This could result in a deterioration of the long-term mechanical properties of these adhesives [53]. In addition, HEMA has a hydrophilic functional group that can absorb water even after polymerization, which in turn, makes the adhesives susceptible to hydrolysis [54–56]. In accordance with these findings, Bacelar-Sá et al. reported that HEMA-containing adhesives showed poor dentin sealing and greater micro-permeability after 1 year of storage in artificial saliva [57]. This investigation may provide an explanation for this result, i.e., the adhesive layers might be susceptible to hydrolysis under acid conditions and the mechanical properties of the adhesives are insufficient to resist acid action and brushing abrasion. Based on the results of Gluma Self-etch group in the present study, we speculated that the dentin-protective property of desensitizers is material-dependent even if the material is a light-curing agent.

Tooth Coat contains 5% NaF dispersed in a hydrogenated rosin matrix. Hydrogenated rosin has high oxidation resistance and thermal stability characteristics. As a class of renewable polymerizable monomer, it is not soluble in water, but by introducing hydrophilic moieties, the rosin-derived polymers become water soluble [58]. This feature might allow Tooth Coat to penetrate into the dentinal tubule before polymerization. The results revealed the efficiency of Tooth Coat in reducing the dentin permeability. Although the permeability reduction after brush abrasion was significantly different from that measured immediately after application, Tooth Coat showed superior durability among the four desensitizers under the acid and abrasive conditions within the experimental conditions of this study. Although no data is available on the performance of Tooth Coat because the product was developed only recently, it is thought that Tooth Coat successfully forms a protective barrier over the dentin to prevent conduction of stimuli according to the results of the present study. Zhou et al. [59] reported that the high-concentrated fluoride-containing varnishes, both Vanish (5% NaF white varnish with tri-calcium phosphate) and Vella (5% NaF clear varnish with xylitol) are not effective in dentin permeability reduction and should be considered as topical fluoride delivering agents rather than tubular orifice-blocking agents. Interestingly, for Tooth Coat, an exceptional durability of permeability reduction was shown, even after being subjected to erosive and abrasive stress. It is thought that various desensitizers containing NaF may exhibit different properties when the matrix is different. The results could be attributed to the mechanical properties of hydrogenated rosin and penetration ability into the dentinal tubules. In addition, fluoride ions released from Tooth Coat could bind to calcium ions and precipitate calcium fluoride deposits [60], which may cause additional blockage of opened dentine tubules, together with enhanced acid resistance.

A direct correlation with *in vitro* design and *in vivo* oral conditions might be inaccurate during the interpretation of results. It should be acknowledged that *in vitro* conditions differ from *in vivo* conditions in that there is no protective tooth pellicle, or the protective effects of salivary buffering, let alone the artificiality of a tooth surface being in continuous contact with an erosive and abrasive challenge. Indeed, only short-term reduction in dentin permeability was examined in this study. Further studies simulating *in vivo* settings that provide thermal, chemical, or mechanical challenge aging are required for more valid and reliable results.

## Conclusions

Within the limitations of this *in vitro* study, the following conclusions can be drawn:

1. All four desensitizers effectively reduced fluid flow through the dentin.
2. The behavioral characteristics of desensitizers under erosive and abrasive stress varied according to the products.
3. Tooth Coat exhibited excellent sealing ability among the other desensitizers.
